# Phages in Therapy and Prophylaxis of American Foulbrood – Recent Implications From Practical Applications

**DOI:** 10.3389/fmicb.2020.01913

**Published:** 2020-08-11

**Authors:** Ewa Jończyk-Matysiak, Ewa Popiela, Barbara Owczarek, Katarzyna Hodyra-Stefaniak, Kinga Świtała-Jeleń, Norbert Łodej, Dominika Kula, Joanna Neuberg, Paweł Migdał, Natalia Bagińska, Filip Orwat, Beata Weber-Dąbrowska, Adam Roman, Andrzej Górski

**Affiliations:** ^1^Bacteriophage Laboratory, Ludwik Hirszfeld Institute of Immunology and Experimental Therapy, Polish Academy of Sciences, Wrocław, Poland; ^2^Department of Environment Hygiene and Animal Welfare, Wrocław University of Environmental and Life Sciences, Wrocław, Poland; ^3^Pure Biologics, Wrocław, Poland; ^4^Phage Therapy Unit, Ludwik Hirszfeld Institute of Immunology and Experimental Therapy, Polish Academy of Sciences, Wrocław, Poland

**Keywords:** American foulbrood, *Paenibacillus larvae*, honey bee, bacteriophages, endolysin, antibiotic resistance

## Abstract

American foulbrood is one of the most serious and yet unsolved problems of beekeeping around the world, because it causes a disease leading to the weakening of the vitality of honey bee populations and huge economic losses both in agriculture and horticulture. The etiological agent of this dangerous disease is an extremely pathogenic spore-forming bacterium, *Paenibacillus larvae*, which makes treatment very difficult. What is more, the use of antibiotics in the European Union is forbidden due to restrictions related to the prevention of the presence of antibiotic residues in honey, as well as the global problem of spreading antibiotic resistance in case of bacterial strains. The only available solution is burning of entire bee colonies, which results in large economic losses. Therefore, bacteriophages and their lytic enzymes can be a real effective alternative in the treatment and prevention of this *Apis mellifera* disease. In this review, we summarize phage characteristics that make them a potentially useful tool in the fight against American foulbrood. In addition, we gathered data regarding phage application that have been described so far, and attempted to show practical implications and possible limitations of their usage.

## Introduction

The honey bee (*Apis mellifera*) is an important element of natural environment that play a vital role in the process of pollination, and contributes to the improvement and maintenance of flora biodiversity (Morse and Calderone, 2000). Bees’ activity also provides such valuable products as honey, bee pollen, propolis, bee wax, and royal jelly, which are widely used by humans in various industries, including food and diet supplement production, cosmetology, natural medicine and pharmacology. Unfortunately, a significant decrease in the number of bee colonies has been observed worldwide in the last few decades. Much attention has been given to colony collapse disorder (CCD), described as an abnormal phenomenon based on the disappearance of the majority of worker bees in a colony; only the queen, lots of food and a few nurse bees remain in the nest to care for the remaining immature bees (van Engelsdorp et al., 2009; van Engelsdorp and Meixner, 2010). In recent years, honey bees have been exposed in the environment to many adverse factors, which include the chemicalization of modern agriculture, large-scale use of plant protection products, environmental degradation, as well as diseases caused by different pathogens and parasites (Cox-Foster et al., 2007; Dainat et al., 2012). In reality, all of these factors tend to overlap and interact, which means that their synergistic action can cause health problems in bee colonies, such as the abrupt disappearance of worker bees from the colony.

American foulbrood (AFB), caused by *Paenibacillus larvae*, is one of the most infectious, dangerous, lethal and easily spreading diseases of *Apis mellifera* caused by different pathogens and parasites. Despite the name, AFB is classified as a notifiable disease with a worldwide distribution in almost all beekeeping regions in each of the five continents (Alippi and Aguilar, 1998). The causative pathogen has been described by White (1906) as *Bacillus larvae*, a Gram-positive, spore-forming bacterium (Genersch et al., 2006) that can produce even more than one billion spores per infected larva (Shimanuki, 1997; OIE (World Organization for Animal Health), 2018). Infectious spores are transferred within or between colonies by worker bees or by beekeeping practices (Sturtevant, 1932; Lindström et al., 2008a, b). Spores are extremely long-lived (they can survive even more than several decades in honey or on hive equipment (Hasemann, 1961; Genersch, 2010) and resistant to unfavorable conditions, e.g., heat and chemical agents, thus they are very hard to remove (Genersch, 2010). Unfortunately, conventional antimicrobial therapies are only effective for the vegetative forms of bacteria, and so far AFB has proved impossible to eradicate anywhere using all available and allowed methods of treatment and prevention. The use of antibiotics to treat AFB is not a permanent solution due to the production of resistant spores and increase in antibiotic resistance in bacterial cells (Lodesani and Costa, 2005; Alippi et al., 2007); they can also contaminate honey, which could be dangerous for humans consuming this product (Ortelli et al., 2004; Martel et al., 2006; Saridaki-Papakonstadinou et al., 2006; Meeraus et al., 2015; Muriano et al., 2015). This is the reason why antibiotic application in AFB treatment has been banned in most European countries (Genersch, 2010; Forsgren et al., 2018). In many countries, disease control even includes burning of infected colonies that generates huge economic losses. Problems associated with the control and treatment of infected colonies result in a significant decrease in honey bee populations, beekeeping industry and, in consequence, agricultural production all over the world.

Bacteriophages may be a promising solution in the treatment and prevention of AFB spread in honey bees (Tsourkas, 2020). These bacterial viruses are commonly found in the biosphere (Clokie et al., 2011). A recent study has demonstrated that phages (both lytic and temperate) may be part of the honey bee gut microbial community (Bonilla-Rosso et al., 2020), participating in its structure modulation, which affects honey bee health (Deboutte et al., 2020). Phages are natural structures, safe and well-tolerated by higher organisms, including humans, and can also be safe for bees. Phages exhibiting lytic activity cause destruction and decay only of their bacterial host, without disturbing the composition of the natural gut microflora (Cieplak et al., 2018), and thus they undoubtedly can be applied as therapeutics. Phage ability to amplify at the site of infection is their another advantageous feature, which is why they are called “self-dosing.” Furthermore, it has been suggested that phages may be used in the food industry, preventing the spread of pathogenic bacteria, degradation of food products and also promoting safe environment in animal and plant food production (Sillankorva et al., 2012). Isolation of new therapeutic phages for these purposes is a relatively simple, inexpensive, and rapid process. The use of phages in prophylaxis and treatment of bacterial diseases is a targeted method, with high specificity for the host of antimicrobial activity, less expensive and safer than conventional antibiotic treatments (Fernández et al., 2019). What is more, US Food and Drug Administration (FDA) approved a phage preparation as food additives in 2006, with a status of generally recognized as safe (GRAS) (García et al., 2008; Moye et al., 2018).

Knowledge of the potential use of bacteriophages in the fight against AFB is sparse, and a small percentage of studies devoted to this subject contribute to this situation. Therefore, in this article, we present data concerning phage application against *P. larvae* infections.

## American Foulbrood

### Pathogenesis and Epidemiology

Endospores of *P. larvae* are the only direct etiological factor of AFB, whereas, vegetative forms can also be harmful to bees through toxin production (Mahdi and Fisher, 2018). The species *P. larvae* comprises four different genotypes – named ERIC I-IV – based on enterobacterial repetitive intergenic consensus (ERIC) primers (Genersch and Otten, 2003; Genersch et al., 2006) that modulate infection with varying degree of pathogenicity. These types have different phenotypic characteristics, including colony and spore morphology, metabolic capacity, sporulation and virulence level (Neuendorf et al., 2004; Genersch et al., 2005, 2006; Forsgren et al., 2008; Rauch et al., 2009; Saville, 2011; Poppinga et al., 2012). A new ERIC genotype has been recently discovered – *Paenibacillus larvae* ERIC V (Beims et al., 2020). A comparison of the virulence of genotypes is presented in Table 1. Epidemiological studies showed that ERIC I and II are the most frequently isolated genotypes from infected colonies (Alippi et al., 2004; Peters et al., 2006; Antúnez et al., 2009; Loncaric et al., 2009), and these strains usually cause AFB epidemics (Fünfhaus et al., 2018). Each of these genotypes causes specific differences in *P. larvae* virulence, corresponding to the time of killing infected larvae (Genersch et al., 2005, 2006). *P. larvae* with genotype ERIC II are faster, with LT100 (lethal time to 100% population mortality) of approximately 7 days, when compared to members of genotype ERIC I that causes slower larva death (LT100 of approximately 12 days) (Genersch, 2007).

**TABLE 1:** *P. larvae* genotypes and their characteristics (Genersch, 2010; Beims et al., 2020).

Genotype	ERIC I	ERIC II	ERIC III	ERIC IV	ERIC V
Species	*P. larvae*		*Paenibacillus larvae subsp. pulvifaciens*		*P. larvae*
Virulence	Kills larvae within 12 days	Shows the highest lethality	Kills larvae within 7 days		Kills even after 3 days
Frequency	Most frequent genotype, found throughout the world	Isolated worldwide, especially in Europe	Not identified in recent decades		Identified in Spain

American foulbrood only affects the initial stages of bee development. Bees exhibit hygienic behavior which includes innate and hereditary behaviors associated, for example, with effective removal of sick/damaged brood by bees to prevent the emergence, spread and transmission of diseases of adult bees and brood. Certain studies demonstrated that some bees, which presented higher hygienic behavior, could better control brood disease, including AFB infection in colony conditions (Palacio et al., 2000; Spivak and Reuter, 2001). Chen et al. (2000) showed that a dose of spores used to inoculate *A. mellifera* and *A. cerana* larvae of the same age would cause 95% mortality in the former case and only 47.1% mortality in the latter. As regards *A. cerana*, lower levels of infection were caused by the removal of 82.2% inoculated larvae by adult bees before reaching the capped stage. However, it is difficult for bees to completely overcome the infection caused by *P. larvae* due to the high infectivity of *P. larvae*, the ability of bacteria to produce spores, as well as the spreading pathway, proliferation and the fact that no symptoms are noticed at the initial stage of infection.

American foulbrood usually spreads horizontally, but can also spread vertically, when colonies swarm (Fries et al., 2006). Horizontal transmission is observed when spores are distributed by adult bees within and between colonies, facilitating spreading of the disease to healthy larvae and colonies (Fries et al., 2006; Poppinga and Genersch, 2015). Another way of AFB spreading is by robber bees, which prey on colonies weakened by AFB infection and may take contaminated honey back to their hives and, as a consequence, spread the disease to other colonies and apiaries. Beekeepers can also be a vector through unintentionally using the same equipment for sick and healthy colonies (Lindström et al., 2008a, b; Pentikäinen et al., 2008). Vertical transmission is observed in honey bees during reproductive swarming (Fries and Camazine, 2001; Fries et al., 2006).

Honey bee larvae become infected during feeding with food contaminated with spores by adults nestmates (Yue et al., 2008). The susceptibility of larvae to disease caused by *Paenibacillus larvae* decreases with increasing age. Larvae until 12–36 h after hatching are most vulnerable to infection. During this time to successfully initiate infection larvae needs to consume a dose of 10 or less spores (Woodrow, 1942). Hansen and Brødsgaard (1999) showed that the mean infective dose needed to initiate infection in 24–28 h-old bee larvae is 8.49 ± 1.49 spores. Larvae older than 48 h become more resistant to infection so that no significant correlation of dose and mortality was observed after this time. The relationship between dosage and mortality is highly dependent on larval age, genetic constitution and bacterial strain (Genersch et al., 2005).

**FIGURE 1:**
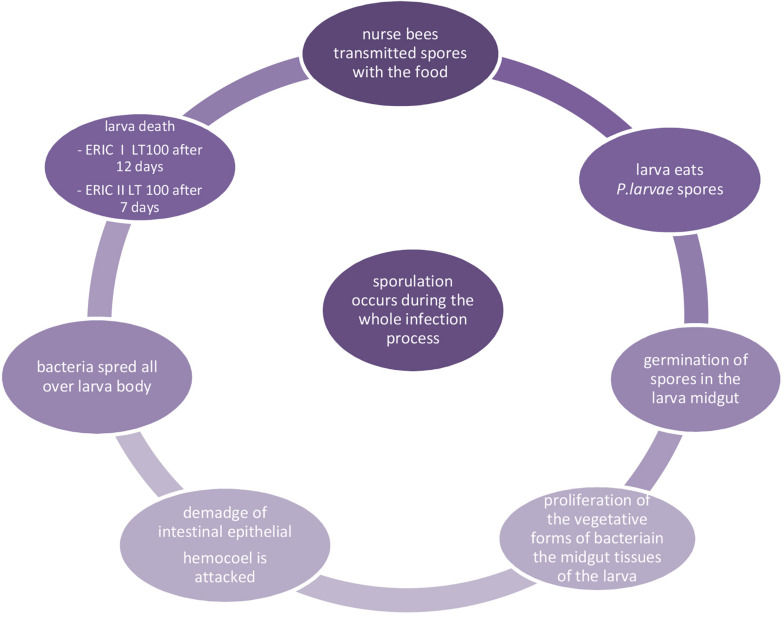
Infection cycle of AFB (Woodrow, 1942; Genersch et al., 2006; Yue et al., 2008).

Once the spores reach the gut of a larva, they germinate and the vegetative forms of bacteria move into the gut tissues, where they multiply. After intestinal epithelial damage and invasion of the hemocoel infected larvae die after their cells are sealed and millions of infectious spores form in their remains. The AFB infection cycle is presented in Figure 1. Dried larval remains adhere to the cell walls and cannot be easily removed by bees, and thus the comb remains contaminated and is a source of spores that can spread within and between colonies. The lifecycle of *P. larvae* in honey bee can be divided into two stages. The first one is the time when spores germinate in the larval midgut, where the vegetative bacterial cells massively proliferate for several days without destroying the epithelial integrity and live on food ingested by the host (Yue et al., 2008). During this period, *P. larvae* metabolize different sugars, which are compulsory to support vegetative growth, by enzymes of the Embden-Meyerhof-Parnas, pentose phosphate, and Entner-Doudoroff pathways involved in carbohydrate metabolism (Julian and Bulla, 1971; Neuendorf et al., 2004; Djukic et al., 2014). During the second stage, the midgut epithelium is penetrated and the hemocoel is attacked by bacterial vegetative cells, which is synonymous of death of the larvae and destruction of larval remains (Neuendorf et al., 2004; Yue et al., 2008). When nutrients become scarce, the *P. larvae* population undergoes sporulation and the remains become brown and mucilaginous, which is the most characteristic clinical symptom of AFB (Lindström et al., 2008b; Poppinga and Genersch, 2015), known as the rope, because viscous larval remains form a ropy thread when drawn out with a match. This glue-colloid dries down and adheres to the cell wall forming a kind of hard scale consisting of billions of spores, and are highly infectious (Bailey and Ball, 1991; Gregorc and Bowen, 1998). According to Stephan et al. (2020), there is a relationship between spore count and disease and colony development.

### Detection and Treatment of Infected Honey Bees

American foulbrood in many countries is a notifiable disease and is required by law to be reported to relevant government authorities. AFB diagnosis is based on the identification of the etiological agent and the presence of clinical symptoms. Symptoms of AFB disease can be detected during inspection of honey bee colonies. In a healthy colony, the comb cells have a compact structure with brood typical of bees at various developmental stages. While AFB is progressing, the brood structure takes on an irregular appearance due to the presence of dead larvae or pupae in cells. AFB-infected combs are drier, darker, and have a slight foul odor. The cells in the comb have sunken caps (Shimanuki and Knox, 2000; Gliński et al., 2006). Choice of samples for testing depends on whether it concerns a suspicious or diseased honey bee colony or analysis in the context of an AFB monitoring or prevention program. Studies using alternative diagnostic methods showed that analysis of honey and bee samples collected at the entrance to the hive are of limited value, because not all samples (only 86 and 83%, respectively) collected from colonies presenting symptoms of infection were positive. Analysis of bee samples from the space of the brood nest, edge frame or honey chamber is more reliable (Gillard et al., 2008).

Microbiological characteristics, polymerase chain reaction (PCR), biochemical profiling, antibody-based techniques and microscopic identification techniques are most commonly used for *P. larvae* identification. Other methods that may also be used to identify this pathogen are based on testing bacteriophage sensitivity, immunological technique or matrix-assisted laser desorption/ionization time-of-flight mass spectrometry (MALDI-TOF) (Stahly et al., 1999; Schäfer et al., 2014). It is also possible to detect *P. larvae* using microbiome analysis (Erban et al., 2017). Real-time PCR analysis of the 16S rDNA gene of *Paenibacillus* represents an alternative, rapid diagnostic tool (Chagas et al., 2010; Martínez et al., 2010; Alippi et al., 2014). Innovative methods, e.g., identification of endolysin cell binding domain (CBD), which targets *P. larvae*, may be suggested to identify bacterial strains that are the causative agent of AFB (Santos et al., 2019). The World Organization for Animal Health (OIE) has presented a broad outline of various diagnostic methods, but due to differences in sensitivity, the most appropriate of the described methods should be selected (OIE (World Organization for Animal Health), 2018). In addition, there are several selective media for *P. larvae* culture: *Paenibacillus larvae* agar (PLA), (Schuch et al., 2001), MYPGP agar (Dingman and Stahly, 1983), BHIT agar (brain–heart infusion medium supplemented with thiamine) (Gochnauer, 1973), J-agar (Gordon et al., 1973) and CSA (Columbia sheep-blood agar) (Hornitzky and Karlovskis, 1989).

The management of AFB spread reduction relies on different methods: the use of antibiotics, natural products or destruction of infected hives (Genersch, 2010). When the presence of bacteria and first clinical symptoms of AFB are detected, double resettlement can also be applied, but it is effective only when the disease is at an early stage of development (Ritter, 2012). Munawar et al. (2010) investigated the shook swarm method that could also be used for AFB control. The results showed significantly decreased spore load in bee mouths by starving them and shifting them to new, clean hives with new foundation sheets.

Nevertheless, burning colonies that exhibit AFB symptoms is considered the most effective control method to prevent spreading the disease and is usually a legal requirement. These restrictions particularly apply in EU and burning is recommended as the only way to destroy infected colonies (Genersch, 2010; Alippi et al., 2014). However, antibiotics are accepted for prophylaxis and treatment in the United States and Canada (Evans, 2003; Genersch, 2010). This forces the development of alternative, natural strategies for the prevention and control of AFB. Therefore, studies have been published that suggest application of essential oils (Fuselli et al., 2008; Chirila et al., 2011; Maggi et al., 2011; Gende et al., 2014; Kuzyšinová et al., 2014; Santos et al., 2014; Ansari et al., 2015), plant extracts (González and Marioli, 2010; Damiani et al., 2014; Hernández-López et al., 2014; Anjum et al., 2015; Piana et al., 2015), propolis (Antúnez et al., 2008; Bastos et al., 2008; Mihai et al., 2012; Bíliková et al., 2013; Wilson et al., 2015) or probiotics (Alonso-Salces et al., 2017; Daisley et al., 2020). In addition, *in vitro* studies showed antimicrobial activity of royal jelly from different geographical origins against *P. larvae* (Bachanová et al., 2002; Bíliková et al., 2009). Rumanovská et al. (2011) observed some potential for omega 3 polyunsaturated fatty acids in reducing the number of *P. larvae*. Another study showed that *Bacillus subtilis* isolated from honey bee guts and honey samples was able to inhibit *P. larvae* development. Alippi and Reynaldi (2006) detected in their research that other bacteria, e.g., aerobic spore-forming *Bacillus megaterium*, *Bacillus licheniformis* and isolates of *Bacillus cereus* also showed antagonistic effects on *P. larvae*. It was also observed that lactic acid bacteria, such as *Lactobacillus kunkeei* decreased the mortality of the brood infected with *P. larvae* (Arredondo et al., 2017). Unfortunately, the methods listed above target active infection, similarly to antibiotics, but do not destroy *P. larvae* spores.

## Antibiotic Resistance of *P. larvae* Strains

Antibiotics are not fully effective antimicrobials when applied in AFB treatment, they can cause many deleterious effects, and do not destroy *P. larvae* spores; they treat symptoms, but do not cure the disease, because they prevent the multiplication of only the vegetative forms of bacteria (Genersch, 2010). Application of antibiotics may result in an imbalance in enteric homeostasis, e.g., a disturbance in the influence of honey bee gut microbiota on bee metabolism or immune response, and increase the chances of fungal infection (Raymann et al., 2017). Moreover, when used for a long time, they may cause selection of resistant mutants among different *P. larvae* strains (Tian et al., 2012; Alippi et al., 2014), leading to antibiotic ineffectiveness. Resistance genes are encoded by mobile genetic elements; bacterial strains acquire them as a result of horizontal gene transfer through phage transduction (Gómez-Gómez et al., 2019). There are data suggesting that antibiotic resistance genes can remain in the environment even for 30,000 years (D’Costa et al., 2011). Interestingly, these genes can be detected in food and transferred to different ecological niches (Godziszewska et al., 2016). Antibiotic residues were detected in different honey bee products, e.g., honey, wax or royal jelly (Hammel et al., 2008; Lopez et al., 2008; Bargańska et al., 2011), thereby reducing honey quality, and potentially affecting the vitality and longevity of bees. A serious threat to humans may be associated with the possibility of antibiotic residue accumulation in commonly consumed bee products (Ortelli et al., 2004; Saridaki-Papakonstadinou et al., 2006).

The World Health Organization (WHO) indicated antibiotic resistance as one of ten biggest threats to global health (World Health Organization (WHO), 2019). Antibiotic application causes the possibility of resistance development in *P. larvae*, and it has already been detected both in the United States, Canada, and Argentina isolates (Miyagi et al., 2000; Evans, 2003; Alippi et al., 2007). It may be acquired, e.g., via genetic transfer (by mobile genetic elements, e.g., plasmids) even between different bacterial genera from soil, and plasmids encoding antibiotic resistance genes were detected, e.g., in commercial honey (Alippi et al., 2014). Wild strains of *P. larvae* were proved to carry oxytetracycline resistance genes (Alippi et al., 2007). It has been demonstrated that resistance of wild *P. larvae* strains may reach even 58% of the samples (Alippi et al., 1999; Miyagi et al., 2000; Spivak, 2000; Murray and Aronstein, 2006; Mitrano et al., 2009). Elzen et al. (2002) reported that macrolide Tylosin Tartrate was more effective in controlling oxytetracycline-resistant *P. larvae*, with no effect on adult and larval bee mortality.

In the United States, antibiotics are permitted in the elimination of *P. larvae*. For decades, oxytetracycline was the only approved antibiotic used for this purpose. But since 2005, the FDA has approved four new products to control the disease. For example, the second antibiotic, tylosin tartrate (TYLAN, TYLOVET, TYLOMED-WS), was approved in 2005, whereas the newest antibiotic against AFB – lincomycin hydrochloride (LINCOMIX) – was approved in 2012 (FDA (Food and Drug Administration), 2020).

In the past, antibiotics and sulfonamides were used in EU in the treatment of colonic diseases. Current legislation (Regulation EEC2377/90 and amendments) prohibits the use of antibiotics and does not allow the presence of their residues in honey and hive-derived products, which prevents their application and, in consequence, limits the range of available methods to fight AFB.

## *P. larvae* Bacteriophages and Their Characteristics

**FIGURE 2:**
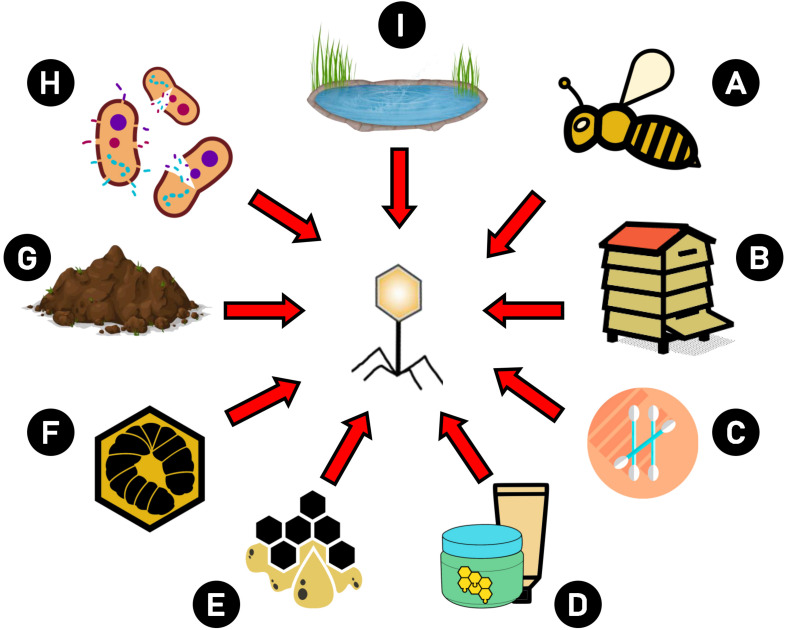
Possible sources of *P. larvae* phage isolation. Phages intended to use against AFB may be isolated from different sources: **(A)** bees and bee products, **(B)** hive elements (e.g., combs), **(C)** swabs from hive area, **(D)** cosmetics which contain bee products, **(E)** wax, **(F)** larvae, **(G)** soil, **(H)** lysogenic bacteria, **(I)** water.

Because an increase is observed in the frequency of antibiotic resistance in bacteria, and stringent regulations prohibiting the use of antibiotics in bee disease treatment, phages are suggested as components that may be intended to combat microbial resistance (NIH, 2014) as effective and safe agents in AFB treatment and prevention. Bacteriophages are naturally occurring bacterial viruses that can be found in the hive and honey bee organisms. Phages active against *Bacillus larvae* were isolated for the first time by Smirnova (1953) from bee larvae suffering from AFB. The source of phages can be: lysogenized bacteria (Gochnauer, 1955; Dingman et al., 1984), water, soil from the hive area (Popova et al., 1976; Valerianov et al., 1976; Ribeiro et al., 2019b), swabs from hive surfaces, beehive materials (Beims et al., 2015) wax, brood (Oliveira et al., 2015), larvae, adult workers and even cosmetics, e.g., containing honey as an ingredient (Merrill et al., 2014; Stamereilers et al., 2016; Yost et al., 2016, 2018; Walker et al., 2018; Tsourkas, 2020). They may be present in the material in which the host bacteria were isolated. Figure 2 presents possible sources of *P. larvae* phage isolation. The phages showed high specificity for *P. larvae* and both lytic (Yost et al., 2016) as well as temperate phages were isolated (Dingman et al., 1984). According to some of the recent data, all so far described phages in *P. larvae* are lytic *in vitro* (Stamereilers et al., 2018), including those induced from prophages, while other study has indicated that they are all temperate (Tsourkas, 2020). Therefore, this classification may cause incompatibilities, and researchers should be very cautious, for example because phages firstly identified as lytic may prove to be induced from bacteria (i.e., they carry integrase genes in their genomes) after detailed analysis, including BLAST (Stamereilers et al., 2018). Strictly lytic phages are safe when applied in phage therapy, because they do not have the possibility to incorporate into the bacterial genome and transduce bacterial genes when compared to temperate ones (Górski et al., 2020). Temperate phages, especially those capable of transferring antibiotic resistance genes, are not safe and should be excluded from phage therapies. However, there are data suggesting that temperate phages could potentially find use in therapy (Chung et al., 2012; Meader et al., 2013), especially in the fight against AFB, as presented by Ghorbani-Nezami et al. (2015). Of course, for safety reasons, their application should be carefully considered. For example, inability to transduce should be proved at the gene level, as in the study of Ribeiro et al. (2019b) on vB_PlaP_API480, and only then phage application potential can be assessed and confirmed *in vivo*.

Morphologically, *P. larvae* phages were mostly identified as an elongated-capsid siphovirus and round-capsid siphovirus (Merrill et al., 2014), but myoviruses were also found (named Abouo, Davies, Emery, Jimmer1, and Jimmer2). The first group of siphoviruses contains phages Diane, Fern, Hayley (Stamereilers et al., 2016), BLA, PBL1, and PPL1c (Merrill et al., 2014). These phages have long, non-contractile tails and elongated capsids. The second siphovirus group contains only PBL3, which have a round capsid (Campana et al., 1991; Merrill et al., 2014). The size of siphovirus phages is approximately 300 nm; phage capsid is approximately 100 nm-long and 50 nm-wide, and tails are approximately 150–200 nm in length (Stamereilers et al., 2016). *Myoviruses* such as Abouo, Davies, Emery, Jimmer1, and Jimmer2 are similar in size to siphoviruses, with an average capsid height of 67.2 ± 3.2 nm and an average width of 64.1 ± 2.6 nm. The average tail length is about 122.0 ± 27.3 nm (Merrill et al., 2014). Ribeiro et al. (2019b) reported isolation of a *Podoviridae* phage, vB_PlaP_API480, with activity against *P. larvae*, characterized using transmission electron microscopy as a phage with an icosahedral capsid and a short 12 × 8 nm non-contractile tail, 58 nm in diameter.

### Genetic Characteristics of Sequenced *Paenibacillus larvae* Bacteriophages

Phages specific to *P. larvae* were already identified in 1950, but genome sequencing was not possible at the time (Smirnova, 1953; Gochnauer, 1955). Phage phiIBB_Pl23, isolated in Portugal by Oliveira et al. (2013), was the first bacteriophage whose genome was fully sequenced. The following five phages: Abouo, Davies, Emery, Jimmer1, and Jimmer2 were isolated and sequenced in Utah, United States also in 2013 (Sheflo et al., 2013). They were first identified as phages specific to *Bacillus larvae*, however, after reclassification of the host bacterium (Genersch et al., 2006; Merrill et al., 2014), the names of phages were changed as specific to *Paenibacillus larvae*. Some publications considered these phages as *Paenibacillu*s (Merrill et al., 2014), while other works did not classify them to this group of phages (Stamereilers et al., 2016, 2018; Tsourkas, 2020).

A new phage PG1 was identified in 2013, whose sequence was submitted to GenBank, but not published in any journal. After 2 years, phages Diva, Lily, Rani, Redbud, Shelly, Sitara (Carson et al., 2015), and Tripp (Abraham et al., 2016), isolated in North Carolina, were also sequenced. HB10c2 was isolated and sequenced in the same year in Germany (Beims et al., 2015). The next nine phages Diane, Fern, Harrison, Hayley, Paisley, Vadim, Vegas, Willow, and Xenia were isolated and described by scientist from the University of Nevada and Texas (Tsourkas et al., 2015). A thorough genomic sequence analysis of these phages was also performed. Phages were compared with each other and with other sequenced *P. larvae* phages; scientists additionally attempted to identify putative protein functions (Stamereilers et al., 2016). In 2014–2016, a large number of *Paenibacillus* phages was isolated by students from the Phage Hunters course at the Brigham Young University (BYU) (Merrill et al., 2018). In addition, the genomes of four *Paenibacillus* phages were sequenced in the Brigham Young University in 2018 (Yost et al., 2018). Stamereilers et al. (2018) analyzed and classified the genomes of *P. larvae* phages whose sequences were available in GenBank. In our opinion, the group of *P. larvae* phages includes many more sequenced phages and is still growing. Sequences available in GenBank are described as *Paenibacillus* phages, but some also as *Brevibacillus* phages, e.g., Abouo, Davies, Emery, Jimmer1 and Jimmer2; there are also phages without a group name, such as bacteriophage Lily, Sitara, Redbud, Shelly, Rani or Diva.

The genome size of *P. larvae* phages ranges from 35 kb (phage HB10c2) to 58 kb (phage Emery). Most *P. larvae* phages reproduce via lytic cycle on used bacterial strains *in vitro*, including those originally induced from prophages, such as Diane, PBL1c, and Xenia (Stamereilers et al., 2018). *Myoviridae* phages like Abouo, Dives, Jimmer1 and Jimmer2 are very similar to each other. For example, Jimmer1 and Jimmer2 share 99.8% average nucleotide identity. Abouo and Daives share 94.9% identity, but Emery differs from other phages (Merrill et al., 2014). Stamereilers et al. (2016) compared nine phages: Diane, Fern, Harrison, Hayley, Paisley, Vidim, Vegas, Willow and Xenia and found that phages Diane, Vadim, Vegas, and Hayley were highly similar to each other; the second similar pair was Harrison and Paisley, and the third: Fern and Willow. Xenia did not seem similar to any other examined phages, but it was found to be similar to phage Shelly isolated by another research group (Stamereilers et al., 2016). Furthermore, scientists classified 17 sequenced *P. larvae* phages into clusters and subclusters based on nucleotide sequence identity; *P. larvae* phages were classified into two main clusters: A and B. Cluster A contained phages Diane, Vadim, Vegas, Hayley, Harrison, Paisley, whereas phages Fern, Willow, Xenia, Diva, Rani, Redbud, Shelly, Sitara, HB10c2 and phiIBB_Pl23 were classified to cluster B. Phage Lily was very divergent from all other *P. larvae* phages and did not belong to any cluster. Cluster B was much more diverse and contained three subclusters and singletons. Similarity between clusters was low, approximately 40%. Differences between clusters included joining protein (gp9), prohead protease (gp10) and partly large proteinase protein. Cluster A phages had identical tail tape measure protein, which was different than the tail tape measure protein of Fern, Willow and Xenia from cluster B (Stamereilers et al., 2016).

**FIGURE 3:**
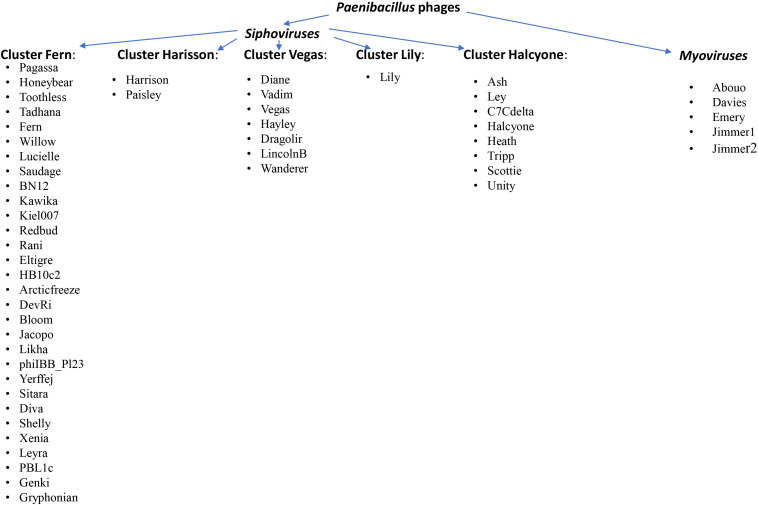
Division of *Paenibacillus* phages into individual groups, including families to which they belong (Stamereilers et al., 2018; Tsourkas, 2020).

After 2 years, the same research group compared 48 sequenced phages against *Paenibacillus larvae* (Stamereilers et al., 2018). They proposed a broader division into five clusters representing the following groups: Fern, Harisson, Vegas, Lily and Halcyone. The Fern cluster was the largest and contained 30 of 48 *P. larvae* phage genomes: Pagassa, Honeybear, Toothless, Tadhana, Fern, Willow, Lucielle, Saudage, BN12, Kawika, Kiel007, Redbud, Rani, Eltigre, HB10c2, Arcticfreeze, DevRi, Bloom, Jacopo, Likha, phiIBB_Pl23, Yerffej, Sitara, Diva, Shelly, Xenia, Leyra, PBL1c, Genki and Gryphonian. The second Halcyone cluster contained eight phages (Ash, Ley, C7Cdelta, Halcyone, Heath, Tripp, Scottie, and Unity), the Vegas cluster included seven phages (Diane, Vadim, Vegas Hayley, Dragolir, LincolnB, Wanderer), while the Harrison cluster contained only two phages: Harrison and Paisley, while phage Lily was a singleton. Phages in the Halcyone cluster were very distant from all other phages. To date, 56 *Paenibacillus* phages have been annotated in GenBank. Supplementary Table S1, which includes characteristics of all phages classified into this group. Figure 3 presents a schematic picture that divides phages into individual groups, including the families to which they belong.

Approximately 90% of *P. larvae* phages show some similarities to other phage sequences from GenBank, about 50% of phage gene products have at least one sequence similarity match to proteins with putative function. The number of genes in the *P. larvae* siphovirus genome ranges from 58 (HB10c2) to 91 (Scottie), with the number of genes changing linearly with genome size (Stamereilers et al., 2018). For myovirus, this number reaches even 102 genes (phages Emery, Jimmer1, and Jimmer2). These similarities enabled the determination of probable protein functions and classification into categories such as structural, assembly, lysis, regulatory, DNA replication and host-related function (Tsourkas, 2020). All *Myoviridae* and *Siphoviridae* phages have a conserved region, located at the start of the genome. This region is located around gp1 to gp17 and codes for virion structural proteins (Stamereilers et al., 2016). Gene products, such as small and large terminase, portal protein, protease and major capsid protein were identified in all phages. Major capsid proteins are encoded by gp5, gp7 or gp8, depending on the phage. This protein builds the phage capsid to the greatest extent. Head-tail joining protein is located at positions gp9 gp10 or gp11. Large terminase (gp2) is involved in DNA packing into empty capsids. Portal protein is involved in the DNA packaging process. Similarity between the architecture of portal oligomers and DNA packaging strategies suggests that portal protein plays the same role in a large number of viruses (Isidro et al., 2004). DNA replication, regulatory and host-related genes are located in the middle of the sequence and usually are not conserved (Tsourkas, 2020). These genes are the most diverse group of genes in phages and they differ significantly between individual phages.

All siphovirus phages encode host lysis genes, usually at position gp21, which codes for a conserved bacteriocin, a toxin produced by prokaryotes inhibiting the growth of competing bacteria (Stamereilers et al., 2016). This gene also has strong BLAST matches to unconfirmed holin-like protein (bhlA protein). *Myoviridae* phages also have holin-like protein but approximately at position gp34-36 (Merrill et al., 2014). DNA regulatory genes form the largest and most diverse group of genes. It consists of genes encoding endonucleases, transposases, integrases, methyltransferases and others. Tail proteins are coded by genes at position gp15-gp16 in all phages. These proteins could have catalytic activity that would allow the phages to enter the host.

## Phage Application Potential

Since the first isolation of phages active against *P. larvae*, many phages were isolated and described, but only some of them were analyzed for their activity in AFB control. Table 2 presents selected data regarding *P. larvae* phage application in bees. The available studies fed infected or healthy larvae with phages or sprayed hive elements, e.g., combs. Ribeiro et al. (2019a) investigated in an *in vivo* study the ability of an active phage to penetrate larvae after *per os* administration of adult honey bees. T7 phage suspension in 50% (w/v) sucrose was applied and phage biodistribution was assessed in adult bees and larvae; phage penetration through food was confirmed in the larval midgut epithelium, which indicated that phages could be active at the site of *P. larvae* infection.

**TABLE 2:** Application of *P. larvae* phage or endolysin in bees.

Applied phages or endolysin	Source of phages	Mode of treatment	Results and recommendations	References
HB10c2 phage	Isolated from environment (the glue-like liquid of a beehive)	Infection during feeding, Bees fed with spores of *P. larvae* strain ERIC I DSM 7030 or ERIC II DSM 2530 at a concentration of 500 cfu/larva, phage was applied at a concentration of 50,000 pfu/larva	Phage did not cause bee mortality and did not disturb gut microbiota composition. However, phage therapy **was not efficient** in AFB treatment in infected larvae	Beims et al. (2015)
F, WA XIII phages	Phages isolated from *P. larvae* strain 2231	Infection during feeding. Larvae were infected with 1000 spores. Single phage (10^5^–10^7^pfu/ml) or phages in cocktail (10^7^pfu/ml) were administered at day 0 or day 1.	Administered phages did not adversely affect survival of larvae. Phages applied before *P. larvae* NRRLB-3650 infection decreased larval mortality; the authors recommend **prophylactic use** of phage therapy against AFB	Ghorbani-Nezami et al. (2015)
PlyPa1A lysin	Isolated from *P. larvae* phage Xenia.	Larvae were infected with *P. larvae* B-3650 spores (1000 spores/larvae) with food were simultaneously treated with lysin at a concentration of 16 μg/ml.	The enzyme was active mainly against genotypes ERIC I. Do not disturb gut microbiota Larvae infected with spores and treated with single dose of the endolysin were rescued in 75%, which indicate the **therapeutic potential**.	Le Blanc et al. (2015)
Cocktail consisted of 7 phages: Xenia, Halcyone, Willow, Fern, Vadim, Harrison and Hayley	Phages isolated from: Xenia-infected hive, Halcyone-propolis, Willow-soil, Fern from wild strain 2231, Vadim- lipbalm, Harrison - soil Hayley- soil	Increasing amounts of food containing cocktail. Application within 7 days. Phage cocktail with a titer of 1.8 × 10^6^pfu/ml was applied before or after infection with spores.	Experiments indicated that **prophylactic administration** of a phage cocktail resulted in a higher survival of larvae than when applied as a treatment.	Yost et al. (2016)
Phage cocktail consisted of three phages (1, 5, 9)	Not known	Phage application with feeding. Phage cocktail applied to uninfected hives, hives in a mock-treated control group with a titer of 10^6^pfu/ml. After 2 weeks, 4 of the 5 hives in the control group were infected with AFB, while the five phage-treated hives remained healthy.	Phages did not cause deaths of healthy bees. The tested phages did not disturb the gut microbiota even after an overdose application and cocktail application, as observed in case of antibiotic application. **Protective and therapeutic effects** were observed in this study.	Brady et al. (2017)
PlyPl23 lysin	Isolated from genome of phage phiIBB_Pl23	Enzyme provided to larvae with feeding (diet containing 2.0 μM of enzyme).	The enzyme is safe and non-toxic for larvae which were observed during 5 days. It did not affect larvae development.	Oliveira et al. (2015)

Unfortunately, phages, similarly as traditional antimicrobials (e.g., antibiotics), have the ability to destroy only vegetative forms of the causative agent of AFB, they are not able to destruct extremely infective spores.

Phage application in apiaries *in vivo* should be preceded by their detailed characterization (phage activity, lytic spectrum, life cycle parameters, genome sequences, phage stability under expected conditions at the site of application or infection) and testing their effectiveness *in vitro.* Ribeiro et al. (2019b) described the API480 phage isolated from a hive soil sample in Spain. The phage showed a broad lytic spectrum and was active against 69% of the tested field *P. larvae* strains *in vitro* representing both ERIC I and ERIC II genotypes. The integrase gene and lysogeny module were not identified in its genome. Examination of phage infection parameters revealed that adsorption was achieved several minutes after phage contact with bacterial cells, 85% of phage particles was adsorbed to its host after 35 min. The latent period lasted approximately 30 min., whereas its burst size was 3 pfu per bacterial cell. Furthermore, the phage was proved to be stable in high 50% (w/v) glucose concentration for 24 h and a slight reduction in phage titer (not statistically significant) was observed in homogenized larvae only after 24 h. These features together with the activity (despite the fact that the phage is temperate) observed *in vitro* suggested that it could be a good candidate for application in hives to treat or protect honey bees in field conditions.

Available studies indicated a possible protective effect for bees infected with these extremely resistant forms of bacteria. The aforementioned effect of three *P. larvae* phages F, WA and XIII was studied *in vivo* by Ghorbani-Nezami et al. (2015) on larvae infected with NRRL B-3650 spores. The authors observed that the survival of larvae treated with phages (phage-treated control) as well as healthy larvae (negative control) was comparable and phages did not cause any deleterious effects. Based on these observations, the authors concluded that applying phages as prophylaxis (before infection with spores) provided better results than using them as therapeutic agents (applied after onset of infection symptoms). However, Brady et al. (2017) showed that phage cocktail active against *P. larvae* may be effective when used both as prophylaxis and therapeutic. Even an overdose cocktail did not exert adverse effects on the mortality of treated bees. The authors compared the efficacy of phage preparations and Tylan Soluble antibiotic, and a 19% decrease in hive health was observed in case of phage treatment applied as a therapeutic, compared to a 38% decrease caused by Tylan. Furthermore, phage application protected hives against *P. larvae* infection in 100%, whereas 80% of untreated hives were infected. These data indicated the potential of phages, especially in the prevention of AFB infection, and showed that a properly composed phage cocktail can be safer and more effective than antibiotics.

### Endolysins and Their Potential Against *P. larvae*

Endolysins are enzymes encoded by bacteriophage genomes and used at the end of their life cycle to degrade peptidoglycan of the bacterial cell wall from within, resulting in cell lysis (Schmelcher et al., 2012). Phage lysins have many advantages, such as high specificity, stability, wide spectrum of activity or high efficiency, which allows their application as effective antimicrobials. Moreover, endolysins do not induce bacterial resistance, therefore, they are considered a promising alternative to phages (Loessner et al., 1995; Schmelcher et al., 2010). Oliveira et al. (2015) described the first *Paenibacillus larvae* endolysin PlyPl23 encoded by the genome of *P. larvae* siphovirus phiIBB_Pl23 with a high lytic potential. The enzyme had an *N*-acetylmuramoyl-L-alanine amidase catalytic domain. Compared to the source of the phage, the enzyme was proved to act specifically and lysed 100% of the tested vegetative forms of *P. larvae*, identified as belonging to different genotypes: ERIC I ERIC II and ERIC III (whereas phage phiIBB_Pl23 lysed only 80% of the tested strains). However, it was not active against *Bacilli* and *Lactobacilli* strains. Due to its high specificity, this enzyme can be applied specifically to eliminate *P. larvae* strains without interfering with bees’ natural microbiota. An *in vitro* study that tested for 7 h heat-activated spores before and after germination showed that both dormant and germinating spores were not sensitive to lysin. It retained the activity in the pH range of 5–9 and after incubation with 25% and 50% sucrose; it was also stable in storage conditions (especially at −20°C for 22 weeks). In addition, lyophilization and reconstitution did not cause a loss of its activity. Interestingly, previous incubation with royal jelly increased the activity of PlyPl23, and a synergistic antibacterial effect between the enzyme and royal jelly was noted. The authors suggested that royal jelly could sensitize the cell wall of bacteria and enhance endolysin activity. *In vitro* lytic activity of the enzyme was also determined by measuring bacterial density in suspension. The authors observed that the activity of 0.2 μM endolysin reduced bacterial density (10^4^ CFU mL^–1^) to non-detectable level after only 30 min. *In vivo* safety tests confirmed that it was not toxic. Le Blanc et al. (2015) isolated lysin PlyPAlA with amidase activity from the genome of *P. larvae* phage Xenia (Le Blanc et al., 2015). Higher enzyme activity *in vitro* was observed against *P. larvae* strain with genotype ERIC I compared to ERIC III and IV. Exposure of *P. larvae* strains to 100 μg/ml of the enzyme reduced bacterial strains viability by 1–2 logs, whereas a dose of 700 μg/ml caused a 4-log decrease, indicating moderate bactericidal activity. Unfortunately, the enzyme did not kill the spores. A slight antibacterial effect was observed only in the case of germinating spores. After applying the enzyme to honey bee larvae, no disturbances were observed in the larval gut microbiota and one dose of lysin rescued up to 75% of larvae infected with spores. The above features indicate that phage lysin seems to be a better candidate than whole phages for preventing and eliminating *P. larvae* infection in bees. Comparison of their amino acid sequences showed high similarity, therefore the described enzymes are probably different variants of one phage protein. It is also possible that the already described *P. larvae* phages may encode previously unidentified lytic enzymes. It has been demonstrated that lysins may specifically bind spores, e.g., lysin LysPBC2 encoded by *Bacillus cereus* (Kong et al., 2019). These findings suggest the probability of isolating an endolysin with activity against spores produced by Gram-positive bacteria similar to *P. larvae*. The results have indicated that there is a need to further search for lysins with the above properties that may be encoded in *P. larvae* phage genomes. The modular structure of these enzymes creates the possibility of engineering proteins and constructing endolysins with new or improved properties. Enhanced lysin activity can be achieved by manipulating their functional domains, e.g., random or directed mutagenesis in the cell-binding domain (São-José, 2018; Kong et al., 2019), shuffling and fusion of catalytic domains with cell-wall binding domains of different origin and properties to obtain chimeric enzymes (chimeolysins), fusion of full-length lytic enzymes, domain deletion, addition or duplication, fusion to peptides, and combination of these methods (São-José, 2018). With respect to AFB sporicidal activity, it would be desirable for tailored enzymes that would penetrate the spore coat and then facilitate bond cleavage in peptidoglycan layers both in the core wall and spore cortex (Todar, 2009).

## Limitations of Phage Use for Elimination of American Foulbrood in Honey Bee

The use of phages or endolysins in the treatment of AFB in honey bee has not only advantages, but also some limitations. Phages are characterized by genomic plasticity. They are able not only to replicate, but also mutate in a specific bacterial host. Additionally, they may induce expression of undesirable virulence factors, toxins and/or antibiotic resistance genes in the host (Łobocka et al., 2014). Most of the isolated phages against *P. larvae*, such as Davies, Jimmer1, Jimmer2, and PG1 are temperate because they encode integrases or transposases, which excludes their possible application in AFB treatment (Merrill et al., 2014; Stamereilers et al., 2018). Phage therapy against *P. larvae*, which is a spore-forming bacterium, may fail because of the possibility of spores protecting bacteria against lysis, which may lead to the development of reinfection (Beims et al., 2015).

Bacteria may already be resistant to phages. This situation means that bacterial susceptibility to selected bacteriophages should be tested every time whenever phages are planned to be utilized against these bacteria. An easier and faster solution is to prepare a cocktail that contains phages of different lytic spectra that can be active against wider host ranges, and application of this type of formulation may limit the probability of acquiring resistance to the applied phages (Merabishvili et al., 2018). Furthermore, bacterial collections on which the preparation activity will be tested should be regularly renewed with pathogenic strains from the area where phages are planned to be used to ensure that the phage preparation is active against currently or locally occurring bacterial strains (Merabishvili et al., 2018). Another possibility is to try to isolate new phages from materials from which pathogenic bacterial strains (that caused AFB) are isolated or various environmental samples that are collected.

There are many factors that may interfere with phage activity (when phages are intended as therapeutics), such as physico-chemical conditions, host physiological conditions, preparation composition or phage inability to penetrate and achieve high concentration at the site of infection (Jończyk-Matysiak et al., 2019). Phage efficiency may depend on its properties, structure and biology as well as therapeutic expectations, dose, manner of application, as well as modifications that can improve their activity and availability. The individual stability of the phage at different pH conditions is also an important issue, and this feature should be checked for each phage, as they tend to have different sensitivities to various physico-chemical conditions (Jończyk-Matysiak et al., 2019). These features should be taken into account and all phages contained in the preparation should be fully characterized. In addition to phages present in the preparation, it is also important to select proper additives that would protect phage activity and act as stabilizers. Moreover, the entire composition of the preparation intended for use in hives or to feed bees should be well tolerated by bees (taste and safety) and contain as few ingredients as possible.

Penetration of phages to the honey bee gut – the site of *P. larvae* infection – should be tested to ensure that the phages penetrate and are active at the site where AFB etiological agent is present. Therefore, spraying hive elements seems to be a less effective route of phage administration, resulting in a lower phage concentration on the hive surface. Despite the confirmed phage penetration at the site of infection, limitations of their action may be associated with low phage concentrations (as observed by Ribeiro et al., 2019a), not sufficient to reduce the count of *P. larvae* to prevent and cure AFB. Therefore, a high dose of phage particles should be provided and a method of phage protection against harmful hive-derived conditions is required (e.g., temperature, humidity, pH of larval food, persistence on the surface of hive elements). That is crucial to retain phage activity, as the conditions listed above vary in hive throughout the year. Bees have mechanisms to control the nest climate and specifically the brood area, but they depend on the prevailing weather conditions outside the nest and bee colony metabolism, which changes during the year (Stabentheiner et al., 2003, 2010; Tautz, 2008; Shaw et al., 2011; Cecchi et al., 2020). Moreover, phage preparations applied in the winter can remain deposited in wax patches even for several months and can cause significant reduction in phage activity. Under varied temperature conditions, phages may lose their activity during persistence in wax patches (Weinbauer, 2004); therefore, in order to protect phages applied in hives to control AFB, methods harmless to bees prolonging phage activity, such as encapsulation providing protection against harmful external factors, chemical or genetic modifications enabling extended phage activity, and addition of stabilizers protecting phages against their titer reduction should be considered.

It was demonstrated that phages could be inactivated during storage in larval food for a long time (Gochnauer, 1970). It was suggested especially in the case of larvae fed with phages and royal jelly that phage inactivation could be caused by the latter (low pH, composition), which could be one of the possible causes of failure of phage therapeutic effect in bees suffering from AFB (Yost et al., 2016; Ribeiro et al., 2019a). Hence, phages should be taken by larvae as fast as possible to ensure a neutral pH in gut conditions that guarantees phage stability. In addition to royal jelly, honey was proved to completely inactivate phages *in vitro* (Oliveira et al., 2017, 2018), which indicated that hive-derived products could influence phage activity in the hive environment.

Although *in vitro* studies on phages or their lysins are promising, phage activity depends on hive environment, larval gut conditions, phage individual features and its stability may significantly limit the effects of phage application *in vivo*. To protect phages and lysins against inactivation in hive and maintain phage stability during and after phage application, different methods may be used, e.g., encapsulation, addition of stabilizers, chemical or genetic modifications (mentioned in section “Endolysins and Their Potential Against *P. larvae*”) to achieve prolonged activity or render phage or its protein extremely insensitive to environmental conditions.

Lysins appear to be effective against specific bacteria. However, studies using *P. larvae* phage lysins are scarce, which makes it difficult to infer from them meaningful conclusions. Since 2015, two lysins have been tested that (similarly to phages) were neither active against dormant nor germinating spores (Le Blanc et al., 2015; Oliveira et al., 2015). More studies should be conducted on a larger number of phage lytic enzymes in both laboratory and hive conditions, especially to allow comparison of the effects that form the full range bactericidal activity of *P. larvae*-encoded lysins. Moreover, studies should be undertaken to develop methods allowing to make lysins more effective (especially against spores), active at the site of infection – in the larva’s gut.

Currently, the trend of using natural products continues both in the treatment of bacterial infections and as part of a diet. Research by Naanwaab et al. (2014) showed that consumers declared to pay extra for bacteriophage-treated fresh product if it would improve their food safety. This indicated that consumers were not afraid of phage application in food, which can suggest that phage residues in bee products may also be acceptable.

Therefore, further research on both bacteriophages and lysins in the fight against AFB is required. Research in this area is still very limited and is urgently needed to save billions of honey bees all over the world.

## Conclusion and Perspectives

There is an urgent need for a safe, natural and effective product for the prevention and treatment of AFB in honey bees, whose application would not cause any adverse effects. There have been reports of isolation of bacteriophages active against *P. larvae*, and attempts of their use in AFB prevention and treatment. Published data indicated that the isolated specific phages showed the ability to lyse only the vegetative forms of *P. larvae* strains, and *in vivo* effects suggested that phages could be particularly useful in AFB prevention rather than treatment. Studies on phage-encoded endolysin are also promising. The use of phages or their enzymes in AFB therapy may reduce the need to eliminate hives by burning. Further research on phage application using different phage titers, different phage formulation compositions, forms of preparation (lysate or purified preparation), application (feeding, spraying), addition of different carriers both *in vitro* and *in vivo* during different stages of bee development is required. In addition, their application may reduce the risk of negative influence on the health of bees and consumers of bee products. Therefore, the possibility of using phages in treating AFB can bring great economic and environmental benefits as well as advantages for human health.

## Author Contributions

EJ-M conceptualized the study and drafted the main part of the manuscript. EP, BO, KH-S, KŚ-J, NŁ, DK, JN, PM, NB, FO, BW-D, AR, and AG contributed to the parts of the manuscript. EP and NŁ prepared figures. EJ-M, EP, and KH-S prepared tables. AG provided support and conceptual advice at all stages of manuscript preparation. All authors revised the manuscript.

## Conflict of Interest

BW-D and AG are co-inventors of patents owned by the L. Hirszfeld Institute and covered phage preparations. KH-S and KŚ-J were employed by the company Pure Biologics. The remaining authors declare that the research was conducted in the absence of any commercial or financial relationships that could be construed as a potential conflict of interest.
