# Supplementary material for: Phages in Therapy and Prophylaxis of American Foulbrood – Recent Implications From Practical Applications
Source: Front Microbiol. 2020 Aug 11;11:1913. doi: 10.3389/fmicb.2020.01913 (PMC7432437; doi:10.3389/fmicb.2020.01913)
Supplement: Supplementary file 1 [file Table_1.DOCX]

Table 1. Characteristics of the described *Paenibacillus*phages (<https://www.ncbi.nlm.nih.gov/genbank/>)

| No. | | Phage name | GenBank accession no. | Length (bp) | Classification | %GC | ORF’s no. | Nature of phage  Isolation source  Host species |
| --- | --- | --- | --- | --- | --- | --- | --- | --- |
| 1 | *Paenibacillus*phage HB10c2 | | NC_028758.1  (KP202972.1) | 35644 | *Viruses; Duplodnaviria; Heunggongvira; Uroviricota; Caudoviricetes; Caudovirales; Siphoviridae; Sitaravirus.* | 41.8 | 56 | Lytic  Environmental source: glue-like liquid, foulbrood slime  Host: *Paenibacillus larvae* |
| 2 | *Paenibacillus* phage phiIBB_Pl23 | | NC_021865.1 ( KF010834.1) | 41294 | *Viruses; Duplodnaviria; Heunggongvira; Uroviricota; Caudoviricetes; Caudovirales; Siphoviridae; Sitaravirus; unclassified Sitaravirus.* | 40.9 | 68 | Temperate  Environmental source: bee hive  Host *Paenibacillus larvae* H23 |
| 3 | *Brevibacillus*phage Jimmer1 | | NC_029104.1 (KC595515.1) | 54312 | *Viruses; Duplodnaviria; Heunggongvira; Uroviricota; Caudoviricetes; Caudovirales; Myoviridae; Jimmervirus; unclassified Jimmervirus* | 38.11 | 102 | Temperate  Environmental source : soil sample  Host *Brevibacillus laterosporus* |
| 4 | *Paenibacillus* phage Tadhana | | MG727700.1 | 37880 | *Viruses; Duplodnaviria; Heunggongvira; Uroviricota; Caudoviricetes; Caudovirales; Siphoviridae; Sitaravirus; unclassified Sitaravirus* | 42.1 | 65 | Unknown  Isolation source: not available in the database  Host: *Paenibacillus larvae* |
| 5 | *Brevibacillus*   phage Jimmer2 | | NC_041976.1  ( KC595514.1) | 54312 | *Viruses; Duplodnaviria; Heunggongvira; Uroviricota; Caudoviricetes; Caudovirales; Myoviridae; Jimmervirus.* | 38.10 | 102 | Temperate  Environmental source: soil sample  Host *Brevibacillus laterosporus* |
| 6 | *Paenibacillus* phage Likha | | MG727702.1 | 39778 | *Viruses; Duplodnaviria; Heunggongvira; Uroviricota; Caudoviricetes; Caudovirales; Siphoviridae; Sitaravirus; unclassified Sitaraviru* | 41.3 | 65 | Unknown  Environmental source: honeycomb  Host *Paenibacillus larvae* |
| 7 | *Paenibacillus* phage Pagassa | | MG727699.1 | 40035 | *Viruses; Duplodnaviria; Heunggongvira; Uroviricota; Caudoviricetes; Caudovirales; Siphoviridae; Sitaravirus; unclassified Sitaravirus* | 42 | 70 | Unknown  Isolation source: not available in the database  Host: *Paenibacillus larvae* |
| 8 | *Paenibacillus*phage Kiel007 | | MG727696.1 | 37985 | *Viruses; Duplodnaviria; Heunggongvira; Uroviricota; Caudoviricetes; Caudovirales; Siphoviridae; Sitaravirus; unclassified Sitaraviru* | 41.8 | 63 | Unknown  Environmental source: bee debris  Host *Paenibacillus larvae* |
| 9 | *Paenibacillus* phage BN12 | | MG727695.1 | 39485 | *Viruses; Duplodnaviria; Heunggongvira; Uroviricota; Caudoviricetes; Caudovirales; Siphoviridae; Sitaravirus; unclassified Sitaravirus* | 42.6 | 73 | Unknow  Environmental source: bee debris  Host: *Paenibacillus larvae* |
| 10 | *Paenibacillus* phage Saudage | | MH454083.1 | 37962 | *Viruses; Duplodnaviria; Heunggongvira; Uroviricota; Caudoviricetes; Caudovirales; Siphoviridae; Sitaravirus; unclassified Sitaravirus* | 41.9 | 65 | Unknow  Environmental source: bee debris  Host *Paenibacillus larvae* |
| 11 | *Paenibacillus*phage Lucielle | | MH431937.1 | 37947 | *Viruses; Duplodnaviria; Heunggongvira; Uroviricota; Caudoviricetes; Caudovirales; Siphoviridae; Sitaravirus; unclassified Sitaravirus* | 41.8 | 65 | Unknown  Environmental source: dead bees  Host *Paenibacillus larvae* |
| 12 | *Paenibacillus* phage Leyra | | MG727701.1 | 42276 | *Viruses; Duplodnaviria; Heunggongvira; Uroviricota; Caudoviricetes; Caudovirales; Siphoviridae; Sitaravirus; unclassified Sitaravirus.* | 41.4 | 75 | Unknown  Environmental source: bee debris  Host: *Paenibacillus larvae* |
| 13 | *Paenibacillus* phage PBL1c | | MG727698.1 | 40611 | *Viruses; Duplodnaviria; Heunggongvira; Uroviricota; Caudoviricetes; Caudovirales; Siphoviridae; Sitaravirus; unclassified Sitaravirus.* | 41.2 | 79 | Unknown  Isolation source: not available in the database  Host: *Paenibacillus larvae* |
| 14 | *Paenibacillus* phage Willow | | NC_041867.1  (KT361650.1) | 37994 | *Viruses;*  *Duplodnaviria; Heunggongvira; Uroviricota;*  *Caudovirales; Siphoviridae; Sitaravirus.* | 41.9 | 68 | Temperate  Isolation source: not available in the database  Host: *Paenibacillus larvae* NRRL 2605 |
| 15 | *Paenibacillus* phage Fern | | NC_028851.1  (KT361649.1) | 37995 | *Viruses; Duplodnaviria; Heunggongvira; Uroviricota; Caudoviricetes; Caudovirales; Siphoviridae; Sitaravirus.* | 41.9 | 68 | Temperate (conserved to lytic in vivo)*  Isolation source: not available in the database  Host: *Paenibacillus larvae* NRRL 2605 |
| 16 | *Brevibacillus* phage Davies | | NC_022980.1 (KC595518.2) | 45798 | *Viruses; Duplodnaviria; Heunggongvira; Uroviricota; Caudoviricetes; Caudovirales; Myoviridae; Abouovirus* | 39.1 | 94 | Temperate  Environmental source: soil sample  Host: *Brevibacillus laterosporus* |
| 17 | *Paenibacillus* phage Toothless | | MH454084.1 | 38832 | *Viruses; Duplodnaviria; Heunggongvira; Uroviricota; Caudoviricetes; Caudovirales; Siphoviridae; Sitaravirus; unclassified Sitaravirus.* | 42 | 64 | Unknown  Environmental source bee debris  Host: *Paenibacillus larvae* |
| 18 | *Paenibacillus* phage  Honeybear | | MH431935.1 | 40054 | *Viruses; Duplodnaviria; Heunggongvira; Uroviricota;*  *Caudoviricetes; Caudovirales; Siphoviridae; Sitaravirus; unclassified Sitaravirus.* | 41.9 | 66 | Unknown  Environmental source: feral bees  Host: *Paenibacillus larvae* |
| 19 | *Paenibacillus* phage phiERICV | | CP019719.1 | 45618 | *Viruses; Duplodnaviria; Heunggongvira; Uroviricota;*  *Caudoviricetes; Caudovirales; Lilyvirus; unclassified Lilyvirus.* | 42.1 | 65 | Unknown  Environmental source: foul brood  Host: *Paenibacillus larvae* subsp. *larvae* strain belongs to Eric_V genotype |
| 20 | *Brevibacillus* phage Abouo | | NC_029029.1 ( KC595517.1) | 45552 | *Viruses; Duplodnaviria; Heunggongvira; Uroviricota; Caudoviricetes; Caudovirales; Myoviridae; Abouovirus.* | 39.2 | 94 | Temperate  Environmental source: soil sample  Host: *Brevibacillus laterosporus* |
| 21 | *Paenibacillus* phage DevRi | | MH431933.1 | 38520 | *Viruses; Duplodnaviria; Heunggongvira; Uroviricota;*  *Caudoviricetes; Caudovirales; Siphoviridae; Sitaravirus; unclassified Sitaravirus.* | 41.5 | 66 | Unknown  Environmental source: bee debris  Host: *Paenibacillus larvae* |
| 22 | *Paenibacillus*phage  Arcticfreeze | | MH431932.1 | 38518 | *Viruses; Duplodnaviria; Heunggongvira; Uroviricota; Caudoviricetes; Caudovirales; Siphoviridae; Sitaravirus; unclassified Sitaravirus.* | 41.5 | 66 | Unknown  Environmental source: bee sample  host: *Paenibacillus larvae* |
| 23 | *Paenibacillus*phage  Gryphonian | | MH431934.1 | 38541 | *Viruses; Duplodnaviria; Heunggongvira; Uroviricota; Caudoviricetes; Caudovirales; Siphoviridae; Sitaravirus; unclassified Sitaravirus.* | 41.5 | 66 | Unknown  Environmental source: bee debris  Host: *Paenibacillus larvae* |
| 24 | *Paenibacillus* phage Genki | | MH454082.1 | 38540 | *Viruses; Duplodnaviria; Heunggongvira; Uroviricota; Caudoviricetes; Caudovirales; Siphoviridae; Sitaravirus; unclassified Sitaravirus.* | 41.5 | 66 | Unknown  Environmental source: bee debris  Host: *Paenibacillus larvae* |
| 25 | *Paenibacillus*phage Jacopo | | MH454079.1 | 38526 | *Viruses; Duplodnaviria; Heunggongvira; Uroviricota; Caudoviricetes; Caudovirales; Siphoviridae; Sitaravirus; unclassified Sitaravirus* | 41.6 | 66 | Environmental source: infected hive  Host: *Paenibacillus larvae* |
| 26 | *Paenibacillus* phage Bloom | | MH454077.1 | 38519 | *Viruses; Duplodnaviria; Heunggongvira; Uroviricota; Caudoviricetes; Caudovirales; Siphoviridae; Sitaravirus; unclassified Sitaravirus* | 41.5 | 66 | Unknown  Environmental source: bee debris  Host: *Paenibacillus larvae* |
| 27 | *Paenibacillus* phage Kawika | | MH431936.1 | 40769 | *Viruses; Duplodnaviria; Heunggongvira; Uroviricota; Caudoviricetes; Caudovirales; Siphoviridae; Sitaravirus; unclassified Sitaravirus.* | 41.8 | 71 | Unknown  Environmental source: dead bees  Host: *Paenibacillus larvae* |
| 28 | *Paenibacillus* phage Yerffej | | MH431931.1 | 43126 | *Viruses; Duplodnaviria; Heunggongvira; Uroviricota; Caudoviricetes; Caudovirales; Siphoviridae; Sitaravirus; unclassified Sitaravirus* | 40.6 | 69 | Unknown  Environmental source: bee debris  Host: *Paenibacillus larvae* |
| 29 | *Paenibacillus* phage Xenia | | NC_028837.1 (KT361652.1) | 41149 | *Viruses; Duplodnaviria; Heunggongvira; Uroviricota; Caudoviricetes; Caudovirales; Siphoviridae; Sitaravirus; unclassified Sitaravirus.* | 41.5 | 77 | Temperate  Isolation source: not available in the database  Host*: Paenibacillus larvae* NRRL 2605 |
| 30 | *Paenibacillus*phage Vegas | | NC_028767.1 (KT361654.1) | 45653 | *Viruses; Duplodnaviria; Heunggongvira; Uroviricota; Caudoviricetes; Caudovirales; Siphoviridae; Vegasvirus* | 43.6 | 86 | Temperate  Isolation source: not available in the database  Host: *Paenibacillus larvae* NRRL 2605 |
| 31 | *Paenibacillus*phage Eltigre | | MH454078.1 | 38675 | *Viruses; Duplodnaviria; Heunggongvira; Uroviricota; Caudoviricetes; Caudovirales; Siphoviridae; Sitaravirus; unclassified Sitaravirus* | 41.4 | 67 | Unknown  Environmental source: bee debris  Host: *Paenibacillus larvae* |
| 32 | *Paenibacillus* phage Diane | | KT361657.1 | 45653 | *Viruses; Duplodnaviria; Heunggongvira; Uroviricota; Caudoviricetes; Caudovirales; Siphoviridae; Vegasvirus..* | 43.6 | 86 | Temperate (conserved to lytic *in vivo*)*  Isolation source: not available in the database  Host: *Paenibacillus larvae* NRRL 2605 |
| 33 | *Paenibacillus* phage Vadim | | KT361656.1 | 45653 | *Viruses; Duplodnaviria; Heunggongvira; Uroviricota; Caudoviricetes; Caudovirales; Siphoviridae; Vegasvirus* | 43.7 | 86 | Temperate  Isolation source: not available in the database  Host: *Paenibacillus larvae* NRRL 260 |
| 34 | *Paenibacillus* phage Hayley | | KT361655.1 | 44256 | *Viruses; Duplodnaviria; Heunggongvira; Uroviricota; Caudoviricetes; Caudovirales; Siphoviridae; Vegasvirus.* | 43.5 | 84 | Temperate  Isolation source: not available in the database  Host: *Paenibacillus larvae* NRRL 2605 |
| 35 | *Paenibacillus* phage Harrison | | NC_028746.1 ( KT361651.1) | 44249 | *Viruses; Duplodnaviria; Heunggongvira; Uroviricota; Caudoviricetes; Caudovirales; Siphoviridae; Harrisonvirus.* | 40.2 | 84 | Temperate  Isolation source: not available in the database  Host: *Paenibacillus larvae* NRRL 2605 |
| 36 | *Paenibacillus* phage Paisley | | KT361653.1 | 44172 | *Viruses; Duplodnaviria; Heunggongvira; Uroviricota; Caudoviricetes; Caudovirales; Siphoviridae; Harrisonvirus* | 40 | 84 | Temperate  Isolation source: not available in the database  Host: *Paenibacillus larvae* NRRL 2605 |
| 37 | *Paenibacillus* phage LincolnB | | MH454081.1 | 40437 | *Viruses; Duplodnaviria; Heunggongvira; Uroviricota; Caudoviricetes; Caudovirales; Siphoviridae; Vegasvirus; unclassified Vegasvirus.* | 42.3 | 72 | Unknown  Environmental source: bee debris  Host: *Paenibacillus larvae* |
| 38 | *Paenibacillus* phage Wanderer | | MH431930.1 | 40448 | *Viruses; Duplodnaviria; Heunggongvira; Uroviricota; Caudoviricetes; Caudovirales; Siphoviridae; Vegasvirus; unclassified Vegasvirus.* | 42.4 | 72 | Unknown  Environmental source: bee debris  Host: *Paenibacillus larvae* |
| 39 | *Paenibacillus* phage Dragolir | | MG727697.1 | 41131 | *Viruses; Caudovirales; Siphoviridae; Vegasvirus; unclassified Vegasvirus.* | 44 | 69 | Unknown  Environmental source: bee debris  Host: *Paenibacillus larvae* |
| 40 | *Brevibacillus*phage Emery | | KC595516.1 | 58573 | *Viruses; Duplodnaviria; Heunggongvira; Uroviricota; Caudoviricetes; Caudovirales; Myoviridae.* | 41.4 | 102 | Temperate  Environmental source: soil sample  Host: *Brevibacillus laterosporus* |
| 41 | *Paenibacillus* phage Ley | | MH454080.1 | 56465 | *Viruses; Duplodnaviria; Heunggongvira; Uroviricota; Caudoviricete; Caudovirales; Siphoviridae; Trippvirus; unclassified Trippvirus.* | 48 | 88 | Temperate  prophage induction  Host: *Paenibacillus larvae* |
| 42 | *Paenibacillus* phage Ash | | MH454076.1 | 56468 | *Viruses; Duplodnaviria; Heunggongvira; Uroviricota; Caudoviricetes; Caudovirales; Siphoviridae; Trippvirus; unclassified Trippvirus.* | 48 | 88 | Temperate  prophage induction  Host*: Paenibacillus larvae* |
| 43 | *Paenibacillus* phage C7Cdelta | | MH431938.1 | 55774 | *Viruses; Duplodnaviria; Heunggongvira; Uroviricota; Caudoviricetes; Caudovirales; Siphoviridae; Trippvirus; unclassified Trippvirus* | 48 | 87 | Temperate  Environmental source: bee sample  Host: *Paenibacillus larvae* |
| 44 | *Paenibacillus*phage Tripp | | NC_028930.1 (KT755656.1) | 54439 | *Viruses; Duplodnaviria; Heunggongvira; Uroviricota; Caudoviricetes; Caudovirales; Siphoviridae; Trippvirus; Paenibacillus virus Tripp* | 48.3 | 92 | Temperate  likely a temperate phage with the capacity to form lysogens on certain *P. larvae*strains |
| 45 | *Paenibacillus*phage Halcyone | | MH460827.1 | 55560 | *Viruses; Duplodnaviria; Heunggongvira; Uroviricota; Caudoviricetes; Caudovirales; Siphoviridae; Trippvirus; unclassified Trippvirus* | 48.6 | 90 | Temperate  Environmental source: soil  Host: *Paenibacillus larvae* |
| 46 | *Paenibacillus* phage Heath | | MH460826.1 | 55560 | *Viruses; Duplodnaviria; Heunggongvira; Uroviricota; Caudoviricetes; Caudovirales; Siphoviridae; Trippvirus; unclassified Trippvirus* | 48.6 | 90 | Temperate  Environmental source: soil  Host: *Paenibacillus larvae* |
| 47 | *Paenibacillus* phage Scottie | | MH460825.1 | 55990 | *Viruses; Duplodnaviria; Heunggongvira; Uroviricota; Caudoviricetes; Caudovirales; Siphoviridae; Trippvirus; unclassified Trippvirus* | 48.5 | 91 | Unknown  Isolation source: hand cream  Host: *Paenibacillus larvae* |
| 48 | *Paenibacillus* phage Unity | | MH460824.1 | 50316 | *Viruses; Duplodnaviria; Heunggongvira; Uroviricota; Caudoviricetes; Caudovirales; Siphoviridae; Trippvirus; unclassified Trippvirus.* | 49.1 | 78 | Unknown  Environmental source: beehive  Host: *Paenibacillus larvae* |
| 49 | *Paenibacillus* phage vB_PlaP_API480 | | MK533143.1 | 45026 | *Viruses; Duplodnaviria; Heunggongvira; Uroviricota; Caudoviricetes; Caudovirales; Podoviridae.* | 39.2 | 77 | Lytic  Environmental source: soil from hives surroundings in Spain  Host: *Paenibacillus larvae* Pl02-27 |
| 50 | Bacteriophage Lily | | NC_028841.1 (KP296792.1) | 44952 | *Viruses; Duplodnaviria; Heunggongvira; Uroviricota; Caudoviricetes; Caudovirales; Lilyvirus; Paenibacillus virus Lily* | 42.7 | 74 | Temperate  Environmental source: AFB diseased bee hive  Host: *Paenibacillus larvae* ATCC 9545 |
| 51 | Bacteriophage Sitara | | NC_028854.1 (KP296796.1) | 43724 | *Viruses; Duplodnaviria; Heunggongvira; Uroviricota; Caudoviricetes; Caudovirales; Siphoviridae; Sitaravirus.* | 41.6 | 74 | Temperate  Environmental source: AFB diseased bee hive  Host: *Paenibacillus larvae* ATCC 25747 |
| 52 | Bacteriophage Redbud | | KP296794.1 | 37971 | *Viruses; Caudovirales; Siphoviridae; Sitaravirus; unclassified Sitaravirus* | 41.8 | 61 | Temperate  Environmental source: AFB diseased bee hive  Host: *Paenibacillus larvae* ATCC 954 |
| 53 | Bacteriophage Shelly | | NC_041909.1 ( KP296795.1) | 41152 | *Viruses; Duplodnaviria; Heunggongvira; Uroviricota; Caudoviricetes; Caudovirales; Siphoviridae; Sitaravirus* | 41.5 | 68 | Temperate  Environmental source: AFB diseased bee hive  Host: *Paenibacillus larvae* ATCC 9545 |
| 54 | Bacteriophage Rani | | NC_029084.1 (KP296793.1) | 37990 | *Viruses; Duplodnaviria; Heunggongvira; Uroviricota; Caudoviricetes; Caudovirales; Siphoviridae; Sitaravirus.* | 41.8 | 61 | Temperate  Environmental source: AFB diseased bee hive  Host: *Paenibacillus larvae* ATCC 9545 |
| 55 | Bacteriophage Diva | | NC_028788.1 (KP296791.1) | 37246 | *Viruses; Duplodnaviria; Heunggongvira; Uroviricota; Caudoviricetes; Caudovirales; Siphoviridae; Sitaravirus.* | 41.2 | 60 | Temperate  Environmental source: AFB diseased bee hive  Host: *Paenibacillus larvae* ATCC 9545 |
| 56 | *Paenibacillus* phage PG1 | | NC_021558.1 (HQ332138.1) | 37644 | *Viruses; Duplodnaviria; Heunggongvira; Uroviricota; Caudoviricetes; Caudovirales; Siphoviridae.* | 42.4 | 67 | Temperate  prophage induction  host *Paenibacillus glucanolyticus* P073A |

*Stamereilers et al. (2016)
